# Soilless farming system design impacts the diversity and composition of microbiota

**DOI:** 10.1128/aem.01167-26

**Published:** 2026-08-04

**Authors:** Auja Bywater, Aline Novaski Seffrin, Jordan E. Bisanz, Francesco Di Gioia, Jasna Kovac

**Affiliations:** 1 Department of Food Science, The Pennsylvania State University 8082, University Park, Pennsylvania, USA; 2 One Health Microbiome Center, The Pennsylvania State University 8082, University Park, Pennsylvania, USA; 3 Department of Plant Science, The Pennsylvania State University 8082, University Park, Pennsylvania, USA; 4 Department of Biochemistry and Molecular Biology, The Pennsylvania State University 8082, University Park, Pennsylvania, USA; University of Georgia Center for Food Safety, Griffin, Georgia, USA

**Keywords:** nutrient solution, aerobic plate count, microbial load, soilless systems, *Acidovorax*, microbiome, hydroponics, controlled environment agriculture

## Abstract

Controlled environment agriculture (CEA), including soilless farming systems, is expanding to improve food security and resource efficiency. However, little is known about how different soilless farming system designs influence microbial populations that may be relevant to plant health and food safety. This study investigated the effects of soilless system type on microbial load and bacterial community composition in nutrient solution and on bok choy leaves over two growing cycles. Soilless systems, including deep water culture (DWC), Kratky (KR), nutrient film technique (NFT), ebb and flow (EF), and drip irrigation (DI), were evaluated. Significant differences in aerobic plate count (APC) in nutrient solution were observed among system types, with the DI system exhibiting the highest counts across both cycles. Increased nutrient solution pH was negatively associated with APC, whereas temperature did not significantly affect microbial concentrations. APC on bok choy leaves at harvesting was not significantly different by system type. Bacterial community composition in nutrient solution significantly varied by system type, temperature, growing cycles, and sampling day. Microbial alpha diversity also varied significantly by system type. Core microbiota analysis identified *Acidovorax*, *Legionella*, and *Caulobacter* as both core and hub taxa, with *Acidovorax* being the only genus detected across all samples. These findings indicate that microbial dynamics differ among soilless system designs and across growing cycles, suggesting that factors not monitored in this study strongly influence microbial community composition. Furthermore, we identified core and hub bacterial genera warranting further investigation of their function in CEA and their impact on plant health and food safety.

**IMPORTANCE:**

This study demonstrated that microbial load and community composition in hydroponic production varied by soilless system type and between growing cycles. We found that system design had a significant effect on community composition. Furthermore, the community compositions differed between growing cycles, suggesting that factors not measured in this study significantly shaped the community structure. Drip irrigation systems exhibited higher microbial loads compared to other systems. Leaf-associated microbial load showed insignificant differences across systems and growing cycles, likely due to limited contact with nutrient solutions. *Acidovorax* was detected across all samples, warranting further investigation of its role in hydroponic systems.

## INTRODUCTION

Controlled environment agriculture, integrating the use of soilless farming techniques that allow for growing fresh produce without soil, is a rapidly growing agricultural sector. Soilless farming allows for growing food in areas otherwise unsuited for farming, including cities, deserts, and regions with poor soil or limited freshwater resources, which can increase food security globally (1, 2). Several alternative soilless production system designs exist, each differing in substrate utilization and nutrient solution delivery strategies. A few examples include the deep water culture (DWC), Kratky (KR), nutrient film technique (NFT), ebb and flow (EF), and drip irrigation (DI) systems (3, 4). Both the DWC and KR are static soilless systems, with plant roots suspended in a reservoir of nutrient solution, with DWC incorporating an air stone to enhance oxygenation (5, 6). The NFT system utilizes shallow channels with a small pump to circulate the nutrient solution continuously over the plant roots (7). In contrast, EF and DI systems incorporate a solid growing substrate, often a peat-based mix, to support root structure and retain moisture (8, 9). EF operates by periodically flooding and draining the root zone, while DI delivers nutrient solution at set intervals through drippers. While these systems operate differently, they all utilize substrate and water, both of which have been identified as potential sources of bacterial introduction. Additional potential sources of bacteria in these systems include nutrient solution, plants, and human handling (10). Understanding the impact of system design on microbial load and composition is important to help inform management of microbial quality and reduce food safety risk.

Each type of soilless growing system influences the interaction between the roots, substrate, nutrient solution, plants, and microbial populations (11). These unique setups can influence factors like oxygen level, pH, temperature, and nutrient availability, all of which are important growth and survival factors for bacterial populations. Different systems also create unique ecological conditions that shape microbial communities in ways not yet fully understood (12, 13). The microbiota in soilless farming systems can influence critical functions like nutrient cycling, biofilm formation, and suppression or proliferation of various bacteria (13, 14). These microbial processes have direct implications for plant health, yield, and food safety and could be utilized to optimize crop management (12).

Many bacteria found in soilless farming systems exhibit traits that may facilitate their persistence in water-based environments, particularly motility and biofilm formation (15–17). Motility allows bacteria to actively move toward nutrients, colonize surfaces, and interact with plant roots, especially in flowing or aerated systems (18). Plant pathogens, such as *Pythium* spp., are associated with plant disease in these systems, while human pathogens, including *Escherichia coli*, *Salmonella*, and *Listeria*, are linked to foodborne illness from contaminated produce (11). Biofilm formation enables microbial communities to adhere to surfaces and resist environmental stressors, including sanitizers (19, 20). These traits are advantageous for bacteria in soilless farming systems, where continuous nutrient solution flow and an absence of soil create selective pressures favoring bacteria capable of surface attachment and active movement. Biofilm-forming bacteria isolated from an NFT system facilitated the attachment and colonization of *Salmonella* on PVC surfaces, further highlighting microbial food safety risks in soilless farming systems (21). However, improved understanding of microbial community composition across hydroponic systems is needed to guide future work on the functional roles and food safety implications of commonly occurring bacteria.

However, despite the impact of microorganisms on plant health, yield, and food safety, it remains unclear how differences in soilless system design, associated environmental conditions, and system type collectively shape bacterial community load and composition. Therefore, this study aimed to investigate the impact of soilless system type and environmental factors on microbial community load and composition over two bok choy growing cycles.

## RESULTS

### The drip irrigation system had the highest aerobic plate counts across growing cycles

**Fig 1:**
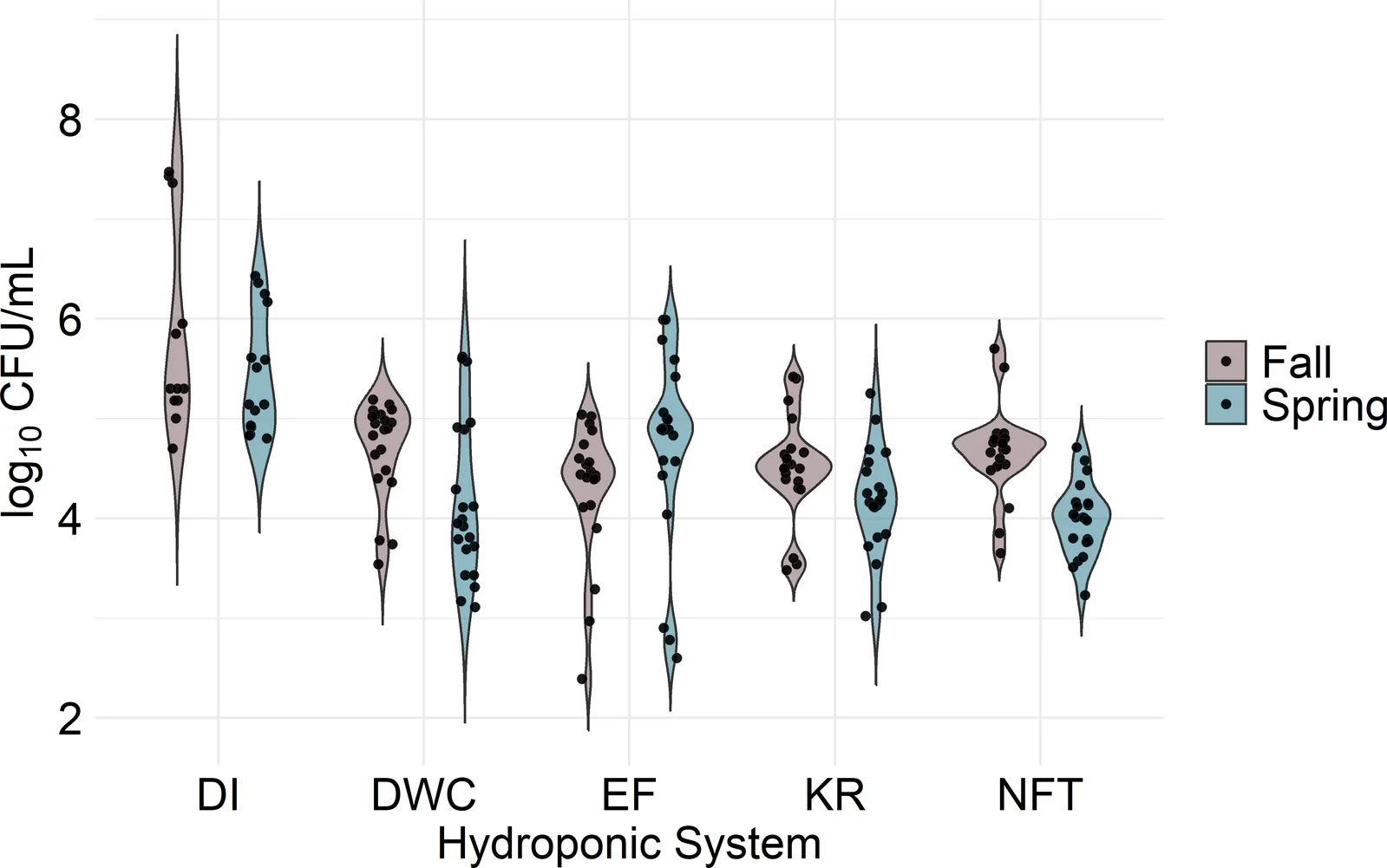
Mesophilic aerobic bacteria concentrations, as measured by APC analysis of nutrient solution sampled from different soilless growing systems in fall (*n* = 97) and spring (*n* = 98) growing cycles. DI, drip irrigation; DWC, deep water culture; EF, ebb and flow; KR, Kratky; NFT, nutrient film technique.

The average aerobic mesophilic bacteria plate counts (APC) in the nutrient solution differed significantly between growing cycles, with higher counts observed in the fall cycle (4.7 ± 0.76 log₁₀ CFU/mL) than in the spring cycle (4.4 ± 0.84 log₁₀ CFU/mL; *P =* 0.001; Fig. 1). Significant differences in APC were observed among hydroponic system types in both cycles (*P* < 1.824 × 10^−8^; Fig. 1). Furthermore, there was a significant interaction between the system and growing cycle (*P* = 0.001; Fig. 1), indicating that the effect of the soilless system type on APC varied by growing cycle. In the fall, APC concentrations ranged from 2.39 log_10_ CFU/mL (EF) to 7.47 log_10_ CFU/mL (DI). In the spring, counts ranged from 2.6 log_10_ CFU/mL (EF) to 6.43 log_10_ CFU/mL (DI). The DI system exhibited significantly higher average APC than the other systems in both growing cycles (*P* ≤ 0.012; fall: 5.9 ± 1.01 log_10_ CFU/mL, spring: 5.44 ± 0.6 log_10_ CFU/mL). Within DI and KR systems, APC did not differ significantly between the two cycles (*P >* 0.08). In contrast, APC was significantly higher in fall than in spring in the DWC (*P =* 0.008) and NFT (*P =* 0.001), whereas the EF system exhibited higher APC in the spring cycle compared to fall cycle (*P =* 0.036). While the system-growing cycle effect on APC in nutrient solution was significant, the impact of the system on APC on bok choy leaves was insignificant in both growing cycles (Fig. S1).

**Fig 2:**
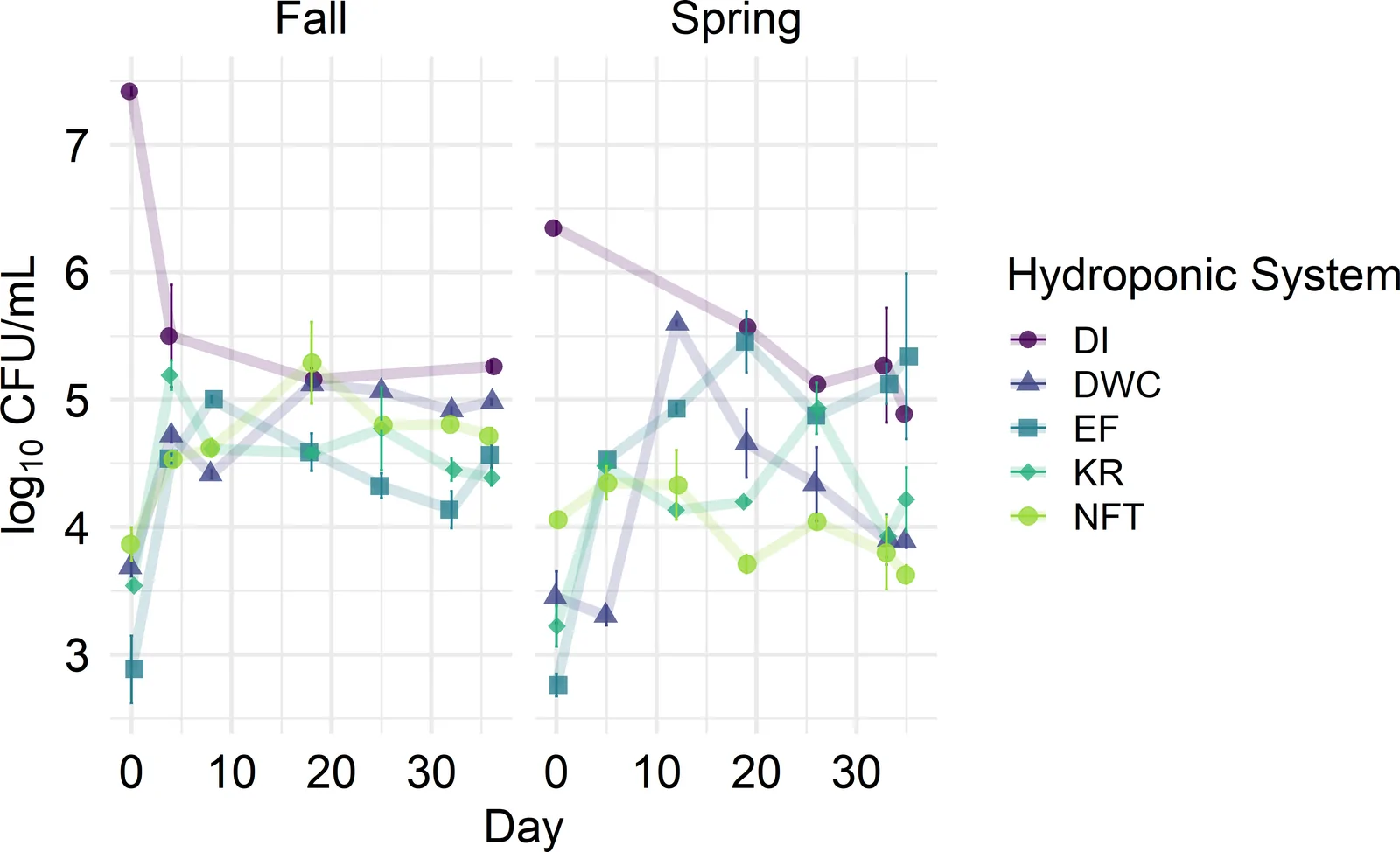
Temporal trends in aerobic mesophilic bacteria concentration, as determined by the APC method, across soilless systems and between growing cycles. The lines connect mean APC values for each system throughout each growing cycle to indicate changes in microbial load throughout the growing cycle. DI, drip irrigation; DWC, deep water culture; EF, ebb and flow; KR, Kratky; NFT, nutrient film technique.

While the DI system consistently maintained the highest APC across both growing cycles, temporal trends varied among systems (Fig. 2). A significant interaction between system and sampling day was observed in both growing cycles (*P =* 5.94 × 10^−9^), indicating microbial levels changed over time and these changes differed by the system. In the fall, APC increased significantly in the NFT (*P =* 0.0171) and DWC (*P =* 0.0002) systems, with concentrations rising from 3.86 to 4.72 in NFT and from 3.69 to 4.98 in DWC. In contrast, the APC in the DI (*P =* 0.0295) systems significantly decreased from 7.42 to 5.26. In the spring growing cycle, the APC in samples collected from the EF system significantly increased over time (*P =* 0.0008), from 2.76 to 5.34, whilethe APC significantly decreased in the DI (*P =* 0.0001) system from 6.35 to 4.89. Temporal changes were not significant in the other tested systems and growing cycles.

### pH influenced APC in the nutrient solution

There were several weeks when the DI system did not have enough nutrient solution run-off to collect a sample for temperature and pH measurements. Due to the missing data, the DI system was removed from the pH and temperature analysis. Furthermore, samples collected on day 0 were excluded from the analysis because the initial nutrient solution temperature used to fill the systems was elevated relative to subsequent time points, confounding the results by correlating higher microbial loads with lower temperatures toward the end of the growing cycle.

**Fig 3:**
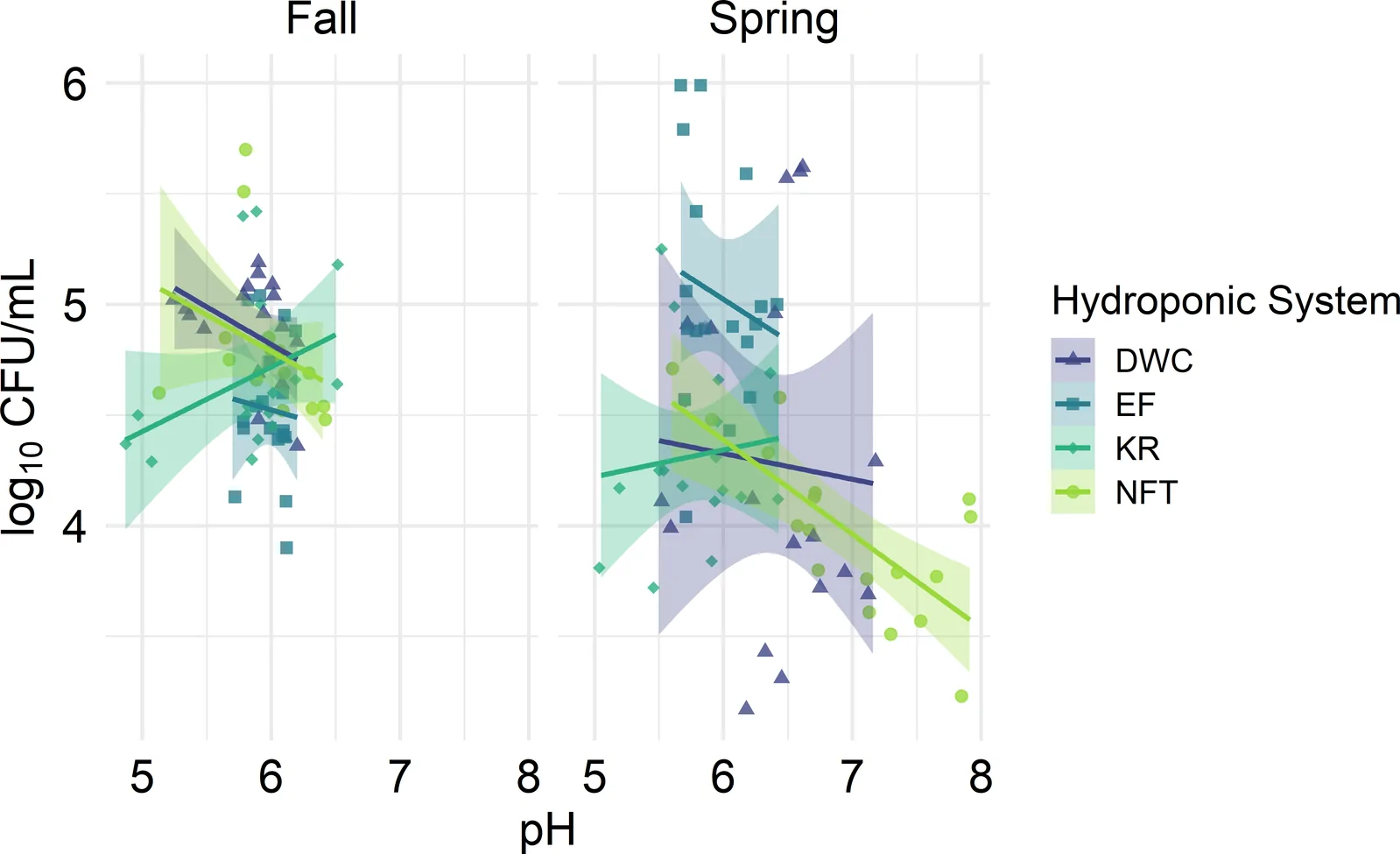
Linear relationships between pH and log_10_-transformed APCs in different soilless systems, faceted by growing cycle. Variation in pH and log₁₀-transformed APCs across soilless systems and growing cycles. Linear regression *R*^2^ values for the relationship between pH and APC counts were as follows in the fall growing cycle: 0.156 (DWC), 0.006 (EF), 0.139 (KR), 0.100 (NFT); and as follows in the spring growing cycle: 0.005 (DWC), 0.032 (EF), 0.012 (KR), 0.543 (NFT). The drip irrigation system is not shown as pH measurements were not possible at all sampling points due to limited run-off in weeks when more nutrient solution was retained by the substrate. DWC, deep water culture, EF, ebb and flow, KR, Kratky, NFT, nutrient film technique.

Nutrient solution temperature differed significantly among soilless systems (*P =* 3.55 × 10⁻⁶) and between growing cycles (*P <* 2.2 × 10⁻¹⁶), with no significant system × growing cycle interaction (*P =* 0.0618), indicating that growing cycle temperature changes were consistent across systems (Table S1). Overall, nutrient solutions were warmer in the spring growing cycle (22.08°C ± 2.65°C) than in the fall (20.4 ± 2.3°C). Nutrient solution pH also varied significantly among systems (*P =* 3.93°C × 10⁻¹⁰) and between growing cycles (*P =* 3.05 × 10⁻⁷) and exhibited a strong system × growing cycle interaction (*P =* 4.17 × 10⁻⁸) (Table S1). Across systems, mean pH was higher in the spring than in the fall growing cycle (6.3 ± 0.7 vs 5.9 ± 0.3) (Fig. 3).

Temperature did not have a significant effect on APC concentration in either growing cycle (*P* = 0.47*)*. In contrast, nutrient solution pH was significantly negatively associated with APC across systems and growing cycles (*P =* 3.45 × 10⁻⁵), with system-specific slope estimates indicating a consistent decrease of approximately 0.36 log_10_ CFU per unit increase in pH across all soilless systems (95% CI: –0.54 to –0.18). This finding may reflect microbial acid production associated with higher bacterial loads. Inclusion of pH in the model reduced the effect of growing cycle, indicating that some of the observed cycle differences in APC may be partially explained by variation in pH. Neither the pH × growing cycle (*P =* 0.26) nor the system × growing cycle × pH interaction (*P =* 0.91) was significant, indicating that the relationship between pH and APC was consistent across growing cycles and soilless systems.

### Bacterial composition differed among soilless systems and growing cycles

Baby bok choy samples were excluded from further analysis due to the high prevalence of chloroplast and mitochondrial sequences resulting from amplification with 16S rRNA V4 primers. For each soilless system and sampling time point, biological replicates were combined prior to DNA extraction. As a result, the data reflect aggregate community profiles, preventing assessment of within-system variability and limiting statistical inference.

**Fig 4:**
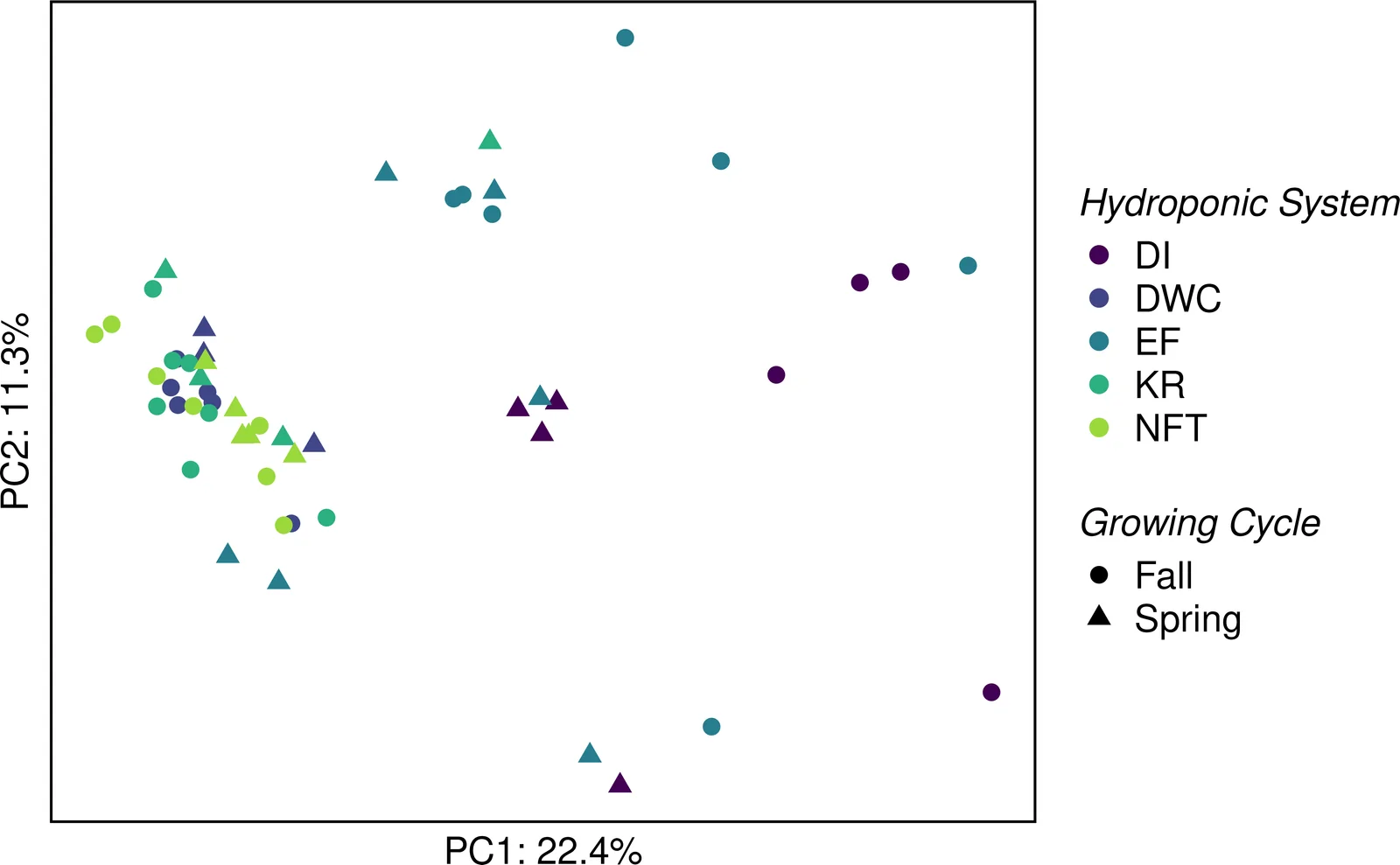
Differences in microbiota composition among systems and between growing cycles. PC1, principal component 1; PC2, principal component 2; DI, drip irrigation; DWC, deep water culture; EF, ebb and flow; KR, Kratky; NFT, nutrient film technique.

Among the factors evaluated, system type was most strongly associated with variation in bacterial community composition in the nutrient solution (*R*² = 0.254, *P =* 0.001), with growing cycle also having a significant, but weaker, contribution (*R*² = 0.053, *P =* 0.001). Their interaction was likewise significant (*R*² = 0.105, *P =* 0.001), indicating that the effect of system type differed between growing cycles (Fig. 4). Sampling day also had a significant effect on bacterial community composition (*R*² = 0.05, *P =* 0.001), indicating that temporal changes within a growing cycle contribute to variation in the microbiota. Unlike APC concentrations, temperature significantly shaped community composition (*R*² = 0.045, *P =* 0.001), whereas pH did not (*P =* 0.125).

The bacterial community composition differed significantly between all systems, with the exception of DWC vs NFT (*P =* 0.26). In the spring growing cycle, fewer differences were observed; DI differed significantly from all other systems (*P <* 0.03), and EF differed from NFT (*P =* 0.01), indicating that growing cycle variation reduced the magnitude of compositional differences among systems.

**Fig 5:**
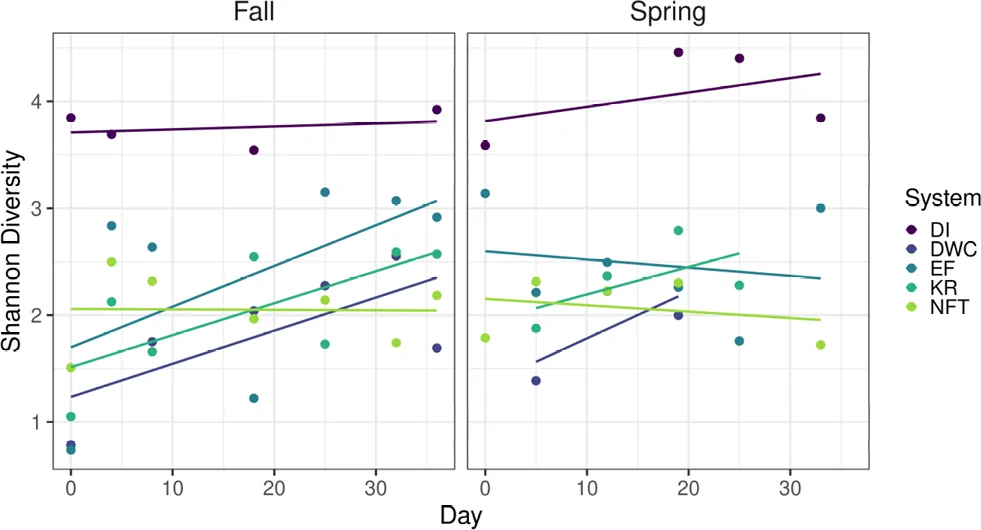
Shannon diversity index temporal trends in soilless systems in fall and spring growing cycles. DI, drip irrigation; DWC, deep water culture; EF, ebb and flow; KR, Kratky; NFT, nutrient film technique.

To assess temporal trends in alpha diversity across different soilless systems during growing cycles, linear regression models were fitted to Shannon diversity index data (Fig. 5). Due to the temperature of the starting nutrient solution being elevated compared to the rest of the samples collected throughout the experiment, we excluded data from day 0 to mitigate confounding results. When data for both growing cycles were combined, the Shannon diversity was not significantly affected by time, pH, nor temperature. System type was the strongest predictor, explaining most of the variation in alpha diversity across samples. In contrast to APC concentrations, pH had an insignificant effect on Shannon diversity (*P =* 0.974), suggesting that while pH influenced the load of mesophilic aerobic bacteria, it did not influence overall community diversity.

### Core and hub taxa were identified across systems and growing cycles

**Fig 6:**
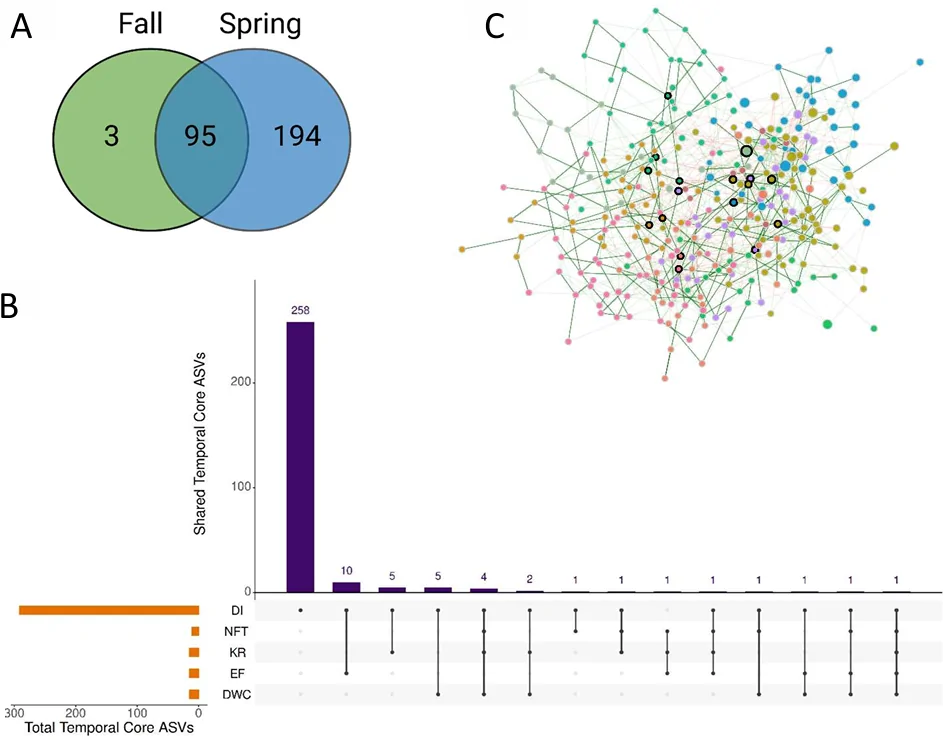
Temporal core ASVs were identified for each system and growing cycles as those present in all samples (i.e., occupancy = 1) collected from one system during one growing cycle. The resulting system- and growing cycle-specific core ASVs were aggregated into a combined set for downstream analyses. Venn diagram shows the number of temporal core bacterial ASVs shared between the two growing cycles, green for fall and blue for spring growing cycle (**A**). UpSet plot shows the number of shared and unique temporal core ASVs among the five systems. Each vertical bar represents the number of ASVs present in the intersection of the systems indicated by the connected black dots below. Horizontal bars indicate the total number of ASVs detected in each individual system (**B**). Networks show bacterial taxa with an occupancy above 0.5 in any system across both growing cycles. Nodes represent ASVs and are color-coded by network cluster, which is a group of taxa that are more strongly connected to each other than to the rest of the network, as determined by the fast greedy algorithm. These clusters represent putative microbial sub-communities that may share similar ecological niches, respond similarly to system conditions, or participate in related metabolic processes. Nodes marked with a black border line are network hubs, determined as those with the highest betweenness centrality, meaning they serve as key connectors or bridges linking different parts of the microbial network. A higher transparency in the edge color indicates lower association value (**C**). DI, drip irrigation; DWC, deep water culture; EF, ebb and flow; KR, Kratky; NFT, nutrient film technique.

Core microbiota were identified to determine which taxa were consistently present in the characterized pooled samples collected from soilless systems and among growing cycles. Core taxa were defined as ASVs detected in all tested samples within a system with no minimum relative abundance. We included one DNA extraction negative control, which yielded 203 reads, suggesting very low-level contamination. None of the ASVs detected in this negative control were identified as part of the core or hub taxa discussed in the paper. The DI system exhibited the highest core richness (292 ASVs), while the DWC, EF, and KR systems each contained 15 core ASVs, and the NFT system contained 11. Despite this variability, one taxon was shared across systems and growing cycles (Fig. 6), suggesting it may be a well-adapted member of hydroponic environments. *Acidovorax* was the only taxon detected in every tested sample and had mean relative abundances ranging from 0.14% (DI, fall) to 7.3% (NFT, fall).

When the occupancy threshold was relaxed to presence in at least 80% across all samples, 20 high-occupancy ASVs emerged as common members of the nutrient solution microbiota. These included both classified taxa, such as *Devosia, Bdellovibrio, Legionella, Rhodanobacter, Sphingobium, Sediminibacterium, Massilia,* and *Caulobacter*, and unclassified taxa affiliated with the *Sphingobacteriales* and *Sphingomonadaceae. Legionella* was represented by multiple ASVs, underscoring the importance of monitoring taxa with potential relevance to water quality.

Our network analysis identified 17 hub ASVs across all samples based on centrality metrics (Fig. 6). These ASVs belonged to the genera *Alicyclobacillus*, *Mycobacterium*, *Acidovorax*, *Dyella*, *Desulfosporosinus*, *Actinoallomurus*, *Chitinophaga*, *Elsterales*, *Nonomuraea*, *Arachidicoccus*, *Peredibacter*, *Legionella*, *Caulobacter*, *Pedobacter*, *Mucilaginibacter*, and *Granulicella*, with *Alicyclobacillus* represented by two distinct ASVs.

**Fig 7:**
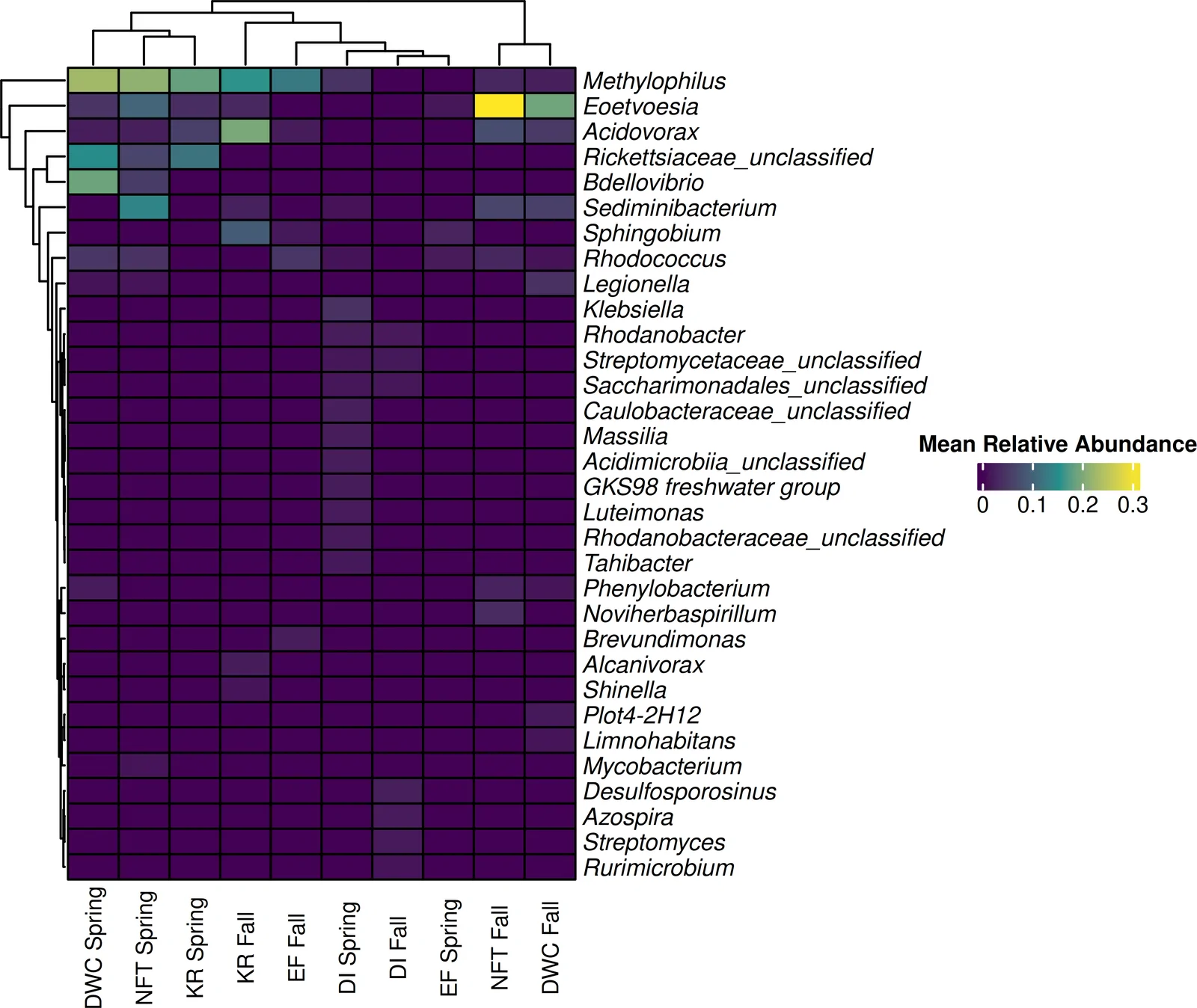
Relative abundance of ASVs averaged over growing cycles for each system. ASVs present in at least 80% of the samples (i.e., occupancy = 0.8) with an abundance greater than 1% are included. DI, drip irrigation; DWC, deep water culture; EF, ebb and flow; KR, Kratky; NFT, nutrient film technique.

Figure 7 shows the relative abundance of ASVs averaged across each growing cycle for all soilless systems. It includes only taxa present in at least 80% of the samples (occupancy ≥ 0.8) and with a mean relative abundance greater than 1%. When systems were averaged by growing cycle, *Methylophilus* exhibited the highest mean relative abundance across every system in the spring growing cycle and remained dominant in the DI and EF systems during the fall growing cycle (Table 1). In contrast, *Eoetvoesia* was the most abundant taxon in the DWC and NFT systems in the fall growing cycle, while *Acidovorax* was most abundant in the KR system.

**TABLE 1:** Genera with highest relative abundances in each system for both growing cycles^*a*^

Soilless system	Growing cycle	Genus	Mean relative abundance (%)	Mean occupancy (%)
DI	Fall	*Methylophilus*	12.9 ± 0.12	75 ± 0.43
	Spring	*Methylophilus*	4.3 ± 0.30	100 ± 0.00
DWC	Fall	*Eoetvoesia*	19.2 ± 0.12	100 ± 0.00
	Spring	*Methylophilus*	22.9 ± 0.23	100 ± 0.00
EF	Fall	*Methylophilus*	12.6 ± 0.12	85.7 ± 0.35
	Spring	*Methylophilus*	18.5 ± 0.23	66.7 ± 0.47
KR	Fall	*Acidovorax*	19.8 ± 0.20	100 ± 0.00
	Spring	*Methylophilus*	18.4 ± 0.07	100 ± 0.00
NFT	Fall	*Eoetvoesia*	30.9 ± 0.24	85.7 ± 0.35
	Spring	*Methylophilus*	21.4 ± 0.13	100 ± 0.00

^*a*^ ASVs with occupancy above 0.5 and abundance above 0.005 were included. DI, drip irrigation; DWC, deep water culture; EF, ebb and flow; KR, Kratky; NFT, nutrient film technique.

## DISCUSSION

### Microbial load and community composition differed by system design and fluid dynamics

The consistently elevated APC in the DI system nutrient solution may be partially attributed to substrate composition. Both DI and EF systems contained a peat-based substrate derived from the surface organic soil layer, composed of partially decomposed plant biomass, which may introduce bacteria and represent a potential source of baseline microbial input. Peat-vermiculite application in soil-based crop production has previously been shown to increase carbon availability and support higher bacterial populations (22). However, because only the DI system exhibited elevated APC across both growing cycles, substrate composition alone does not fully explain the observed differences.

Differences in nutrient solution delivery and retention may have contributed more directly to this pattern. The DI system uses a top-down irrigation approach, in which nutrient solution is repeatedly applied at the surface and percolates by gravity through the entire substrate profile. This flow path increases hydraulic connectivity across the full root zone and promotes detachment and transport of substrate-associated microorganisms into the nutrient solution. In contrast, the EF system operates via periodic bottom-up flooding driven by capillary rise followed by gravitational drainage, which minimizes interaction with the upper substrate layers and potentially restricts microbial mobilization from the full substrate matrix. Together, these hydrological differences are consistent with, and may help account for, the elevated APC observed in DI relative to EF. They may also help explain the sharp APC decline observed in DI after day 0, which may reflect an initial flush of substrate-associated bacteria during first submersion that diminished as the system stabilized.

The same fluid-dynamics framework may help explain why system type was the strongest factor associated with variation in bacterial community composition in our data set (*R*^2^ = 0.254). Bacterial community composition also differed by system in a study of nutrient solution collected from four system types (NFT, DWC, EF, and vertical drip) across 10 commercial facilities in Ohio (13), indicating that community structure likely varies by irrigation architecture across both small-scale and commercial operations, and across different geographic regions. This pattern is not unique to soilless production. In soil-based systems, differences in drip irrigation depth and pattern have been associated with differences in root-soil-microbiome interactions, suggesting that fluid movement is broadly associated with microbial community variation (23). Taken together, these findings suggest that fluid dynamics and irrigation design, more so than substrate identity alone, are likely associated with both microbial load and bacterial community differences across soilless systems.

The APC counts from the bok choy leaves in our study were not significantly influenced by the system, growth cycle, pH, or temperature; likely because they were not in direct contact with the nutrient solution. Other research has reported higher bacterial loads on external leaves in contact with irrigation water in hydroponic systems in Puerto Rico (24). In our study, the outer leaves had minimal or no contact with the water, likely leading to a lack of significant influence.

These findings have practical implications for hydroponic production. Systems that promote extensive contact between water and organic substrates may support higher and more diverse bacterial populations. This may increase opportunities for pathogen introduction or persistence. However, greater microbial competition could also limit pathogen establishment within the system. Overall, these results suggest that differences in hydroponic system design may shape microbial interactions in ways that influence both pathogen risks and the stability of nutrient solution microbial communities.

### Microbial load and community diversity were associated with environmental factors

A notable pattern in our data is that the factors associated with bacterial load were not the same as those associated with bacterial diversity. The nutrient solution pH in our study was significantly negatively associated with APC, with APC decreasing by approximately 0.36 log_10_ CFU/mL for each unit increase in pH across systems. This finding may reflect microbial organic acid production associated with higher bacterial loads (25). Although few studies have directly examined pH effects on APC in soilless systems, previous work reported that increasing nutrient solution pH from 5.0 to 6.5 increased populations of *Clavibacter michiganensis* subsp. *michiganensis* (23). This discrepancy may reflect differences in experimental context, since that study evaluated bacterial survival in nutrient solution alone, whereas our study was conducted in fully operational systems with plants present. A pH-driven shift in metabolic activity may also be at play. Environmental pH changes as small as one unit have been shown to alter microbial community metabolic activity by as much as 50% (26), raising the possibility that pH-associated changes in nutrient availability, which we did not directly measure, partially confounded this relationship. System type also contributed to pH variation. The NFT pH was significantly higher than DWC, EF, and KR, and KR pH was significantly lower than DWC. Although pH was negatively associated with APC within systems, differences in mean APC among systems did not fully correspond to system-level pH rankings. This indicates that while APC and pH have a relationship, other factors not measured in this study likely contributed to the observed system-specific APC differences.

In contrast, pH had an insignificant effect on Shannon diversity (*P =* 0.974), even though it was a strong predictor of APC. Temperature showed the inverse pattern; it had an insignificant effect on APC but was significantly associated with differences in community composition. This decoupling suggests that pH was primarily associated with the amount of bacterial biomass present, while temperature and system type were more closely associated with microbiota composition. A related study of four commercial soilless systems (NFT, DWC, EF, and vertical drip) growing lettuce found that both temperature and pH influenced bacterial variation across leaf, root, growth media, and nutrient solution samples (13). The broader influence of pH in that study may reflect the inclusion of additional sample types and the larger scale of commercial production. Overall, average nutrient solution conditions in our experiment (22.05 ± 2.04°C, pH 6.3 ± 0.63) fell within a range supportive of growth for most environmental- and soilless farming-associated bacteria (19, 27), yet growing cycle, temperature, system, and sampling day each still had significant, and largely independent, effects on community composition. Factors that were not fully accounted for by pH or temperature alone warrant further investigation to inform the management microbial quality and safety in hydroponic production.

### Core and hub taxa reveal distinct ecological roles within the nutrient solution microbiome

The consistent presence of *Acidovorax* across every system and growing cycle underscores its ecological compatibility with soilless nutrient solutions. *Acidovorax* are gram-negative, motile bacteria, and motility support dispersal, surface colonization, and resilience in water-based, low-organic-carbon environments (28). Because organic carbon is typically limited in nutrient solution, heterotrophic bacteria such as *Acidovorax* may be drawn chemotactically toward root exudates, potentially forming biofilms on root surfaces as previously demonstrated (29). Some *Acidovorax* strains are also capable of nitrate-dependent Fe(II) oxidation coupled with organic carbon utilization, a mixotrophic lifestyle that may further facilitate persistence in carbon-limited environments (30, 31). However, these mechanisms remain hypothetical in our system, as we did not measure root exudates or characterize *Acidovorax* phenotypes directly. While several *Acidovorax* species are known plant pathogens affecting cereals and leafy greens (e.g., *A. avenae*, *A. citrulli*, *A. cattleyae*) (32, 33), no disease symptoms were observed in this study, suggesting the strains present were either nonpathogenic or present below disease-causing thresholds. Other *Acidovorax* species are known to be beneficial, contributing to nitrification, phytohormone production, and antibacterial or antifungal activity (34–36).

Many of the other high-occupancy genera we identified have also been reported in hydroponic, aquatic, or plant-associated environments. A study looking at water inputs for hydroponic greenhouses in France found that unclassified *Sphingomonadaceae* had the highest relative abundance in rainwater, while *Devosia* and *Bdellovibrio* were more abundant in groundwater sources (37). *Sediminibacterium* had a relative abundance greater than 1% in more than half of the samples collected from an NFT system growing Brassica leafy vegetables in Singapore (18). *Massilia* and *Rhodanobacter* dominated the rhizosphere of hydroponically grown melons in Taiwan and aquaponic systems in South Africa (38, 39). *Caulobacter* was identified in samples from hydroponically grown plants in China (21). In slow sand filtration systems used for greenhouse water treatment, *Legionella* species have constituted a substantial portion of the resident bacterial community (40).

These high-occupancy genera exhibit several traits that may help explain their persistence across soilless farming systems. Many of them are motile, which likely promotes their widespread distribution in aqueous-based systems (11–13, 41). Several genera identified here are also associated with aquatic or rhizosphere environments and may contribute to functions such as nutrient cycling or microbial regulation (42–44). For instance, *Bdellovibrio* is a known bacterial predator that can influence community structure by targeting gram-negative bacteria (45), while *Devosia* includes nitrogen-fixing and plant-associated species even in the absence of a traditional soil rhizosphere. *Rhodanobacter* has been identified as a bacterial marker of amino acid regulation in hydroponically grown chives and contributes to nitrification, denitrification, and detoxification of plant-derived toxins (46). *Sphingobium* spp. have been implicated in the degradation of polycyclic aromatic hydrocarbons in hydroponically cultivated sudangrass (24). *Caulobacter* may act as a pathogen antagonist in soilless systems, inhibiting wildfire disease, caused by *Pseudomonas syringae* pv. *tabaci*, and other plant pathogens (21, 47). *Devosia* species possess permeases that enhance environmental sensing and chemotaxis (11) and have been isolated as root- and hyphal-colonizing bacteria in mycorrhiza-rich substrates (48). The persistence of unclassified *Sphingobacteriales* and *Sphingomonadaceae* emphasizes the metabolic versatility and biofilm-forming potential of soilless farming microbiomes (49, 50). Our interpretation of the results is based on published literature describing the ecological and metabolic traits of substrate-associated microbiota in hydroponic and plant-associated systems. However, these hypothesized mechanisms were not experimentally validated in the present study and warrant further investigation. Previous work in soil-based systems has shown that drip irrigation delivery methods, like those found in our DI system, can substantially influence microbial composition (51). Wang et al. (23) reported that differences in drip irrigation depths and patterns influenced root-soil-microbe interactions, suggesting that fluid movement plays a key role in selecting for specific microbial assemblages. Although this was a soil-based study, this broader principle supports our finding that differences in nutrient solution flow and distribution across system types can result in distinct bacterial community structures. These observations suggest that fluid dynamics in soilless systems may be an important determinant of bacterial persistence and differentiation.

System design and fluid movement shape which bacteria persist in these systems, and also how bacteria interact with one another within these communities. Hubs are highly connected taxa that play central roles in microbial interactions within a community. The disruption of key taxa can ripple through the community via microbe-microbe interactions and can lead to the collapse of critical ecological functions (52). Unlike abundant taxa, hubs are defined by their extensive connectivity, exhibiting a high degree of betweenness centrality (i.e., being the shortest path between other taxa), linking multiple, otherwise weakly connected taxa. This position in a community suggests that they may mediate resource exchange, signaling, and cross-feeding that enhance community stability and resilience. Hubs often influence nutrient cycling and buffer environmental fluctuations that act as stressors. These taxa are often considered keystone taxa with important ecological roles (53).

Notably, *Acidovorax*, *Legionella*, and *Caulobacter* were identified as both the core and hub taxa, although as different ASVs. This pattern indicates that while these genera are consistently present in the system, different strains may occupy distinct ecological roles. Such ASV-level divergence suggests functional differentiation or ecological specialization within these genera, with some strains acting as highly connected network hubs and others contributing to widespread occupancy.

Functional traits may help explain why many of these taxa serve as hubs. Several genera identified as hubs, including *Alicyclobacillus*, *Mycobacterium*, *Caulobacter*, and *Dyella*, are known to be motile and capable of forming biofilms (54–57). These hubs are also capable of surface colonization and possess metabolic and ecological traits that can support or regulate other microorganisms within the community. For example, through enzymatic conversion of phenolic compounds, *Alicyclobacillus* participates in aromatic carbon degradation, generating metabolites that may serve as substrates for other microorganisms (58). Members of the genus *Acidovorax* exhibit metabolic versatility, including the ability to reduce metal oxides and perform denitrification through both extracellular and intracellular electron transfer pathways (59). *Caulobacter* also exhibits morphological plasticity, forming filamentous cells under environmental stress that can extend above the biofilm surface and enhance nutrient access, dispersal, and resistance to predation (47). These adaptive behaviors can restructure the physical architecture of bacterial communities and contribute to the persistence and functional centrality of *Caulobacter* in soilless farming environments. These traits promote persistence, surface colonization, and interaction with other microorganisms, which likely increases their centrality in network structure.

Integrating relative abundance patterns with occupancy data provided insight into how microbial composition shifts across systems and growing cycles (Fig. S2 to S11). *Methylophilus* consistently dominated communities in the spring growing cycle and remained abundant in DI and EF during the fall growing cycle, whereas *Eoetvoesia* was more prominent in the fall growing cycle communities of DWC and NFT. *Acidovorax* was the prevailing taxon in the KR system during the fall growing cycle. Notably, although *Acidovorax* appeared in both the core and hub taxa lists, *Methylophilus* and *Eoetvoesia* did not, despite their high relative abundance. Although *Methylophilus* and *Eoetvoesia* reached high relative abundance, their absence from the core and hub taxa suggests that these genera may respond opportunistically to shifts in root-derived carbon inputs rather than occupying persistent ecological roles. Root exudation is known to vary with plant growth conditions and environmental context, and changes in exudate composition can rapidly restructure rhizosphere communities (60). Neighbor-induced exudate shifts in soil systems demonstrate how sensitive microbial communities are to plant metabolic signals, and similar fluctuations in soilless systems may explain why some taxa bloom transiently but do not achieve widespread occupancy or network centrality (19, 61).

### Limitations and future directions

Several limitations should be considered when interpreting the results from this study. APC was measured using Petrifilms in the fall growing cycle and standard plate count agar (SPCA) in the spring growing cycle due to a Petrifilm shortage. Although both methods target mesophilic aerobic bacteria, methodological differences may have introduced some variability into cross-cycle comparisons. Furthermore, biological replicates were pooled prior to DNA extraction for each system at each time point, meaning community composition data reflect aggregate profiles for each sampling time point that prevent assessment of within-system variability at a given sampling time point.

More broadly, several of the possible mechanistic explanations proposed here, including root-exudate-driven chemotaxis, biofilm formation, and the ecological roles inferred for core and hub taxa, are grounded in prior literature but were not experimentally validated in this study. We did not measure root exudates, isolate or phenotypically characterize key taxa such as *Acidovorax,* or assess how pH-associated shifts in nutrient solution composition might confound the pH-APC relationship we observed. The significant effect of growing cycle on both APC and community composition, independent of the measured variables, further suggests that unmeasured factors were at play. Future work incorporating nutrient solution chemistry, root exudate profiling, and targeted strain isolation would help test the mechanistic hypotheses proposed here and clarify how irrigation design, growing cycle, and microbial ecology jointly shape microbial community composition and food quality and safety outcomes in hydroponic production.

## MATERIALS AND METHODS

### Experimental location and soilless systems setup

The study was conducted at the Penn State Greenhouse Facility at University Park, PA, comparing, side by side, under controlled environmental conditions, five alternative soilless growing systems, including DWC, KR, NFT, DI, and EF, using bok choy (*Brassica rapa* subsp. *chinensis*) cv. “Li Ren Choi” (Johnny’s Selected Seeds, Winslow, ME, USA) as a test crop. Each soilless growing system was built as a modular system with a footprint of 1.44 m^2^ (1.2 × 1.2 m), and 36 plants per unit were planted, establishing a density of 25 plants/m^2^ across all soilless systems. Soilless systems were arranged in a randomized complete block design with three replications. The study was repeated twice, in the fall-winter growing cycle (November–December) of 2023 and the winter-spring growing cycle (February–March) of 2024.

The alternative soilless systems tested were selected for their popularity and adoption at the commercial scale and for their unique design features. As described by Blunk et al. (3) and Poudel and Di Gioia (4), the DWC systems were set up using large (135 × 135 × 17 cm) black trays (Botanicare, Vancouver, WA) sustained by metallic frames. The trays served as reservoir tanks and were filled with nutrient solution. Styrofoam (expanded polystyrene) floating boards (rafts) were cut to fit each tray. Planting holes, each with a small net pot used to hold in place the bok choy seedlings, were set into each raft in six staggered rows (20 cm apart) to establish the defined plant density (25 plants/m^2^). An air pump (60 L/min, Vivosun, Ontario, CA, USA) with four air stones per module was used to continuously aerate the nutrient solution. The KR system differed from the DWC system, only in two aspects: (i) the styrofoam board did not float on the nutrient solution but rather was suspended at the top of the tray filled with nutrient solution, and (ii) the nutrient solution was not aerated with the air pump; instead, as the nutrient solution was consumed, it was not replenished, and the plants were oxygenated through the air gap created between the surface of the nutrient solution and the styrofoam rafts holding the plants. The modular NFT systems (CropKing Inc., OH, USA) had the same footprint as the other soilless growing systems and were built with six 1.2 m-long food-grade PVC channels, mounted on a metallic frame with a slight slope (4%). Each growing channel had six (2.5 × 2.5 cm) holes 20 cm apart, in which bok choy seedlings were positioned. The nutrient solution serving the NFT systems was kept underneath each module in a 94 L black reservoir tank containing a submersible water pump (Active Aqua, AAPW250, Fairfield, CA, USA) that continually served each growing channel. The drained nutrient solution was recollected in the same reservoir tank by gravity, and the nutrient solution was oxygenated while falling from the PVC collector pipe placed at the lower end of the NFT growing channels into the reservoir tank. The NFT system can be classified as a recirculating hydroponic growing system.

The DI and EF systems were set up using the same large trays and metallic frames used for the DWC systems. However, in this case, bok choy plants were grown in pots (black) of 1.45 L (Koba Corporation, Middlesex, NJ, USA) with holes at the bottom, filled with a peat-perlite growing media mix (PRO-MIX BX, Premier Tech Ltd., Rivière-du-Loup, Canada), and placed in six rows of six pots, establishing the same plant density as the other systems. The EF systems had a 220 L reservoir tank placed underneath each growing tray, and the trays were flooded with nutrient solution using a submersible water pump activated through a timer for 5 min once a day. The nutrient solution was absorbed by the pots through subirrigation, and the excess was drained and recollected in the same reservoir tank underneath each EF growing module.

In the case of the DI system, the setup was very similar to the EF system. However, the nutrient solution was delivered by auto-compensating drippers with a flow rate of 1 L/h. A single dripper served two pots, and fertigation events were scheduled with a timer twice a day. The excess of nutrient solution was drained and collected into a bucket placed at the bottom of each module but was not recirculated. In the EF growing systems, the nutrient solution was delivered from the bottom of the pots and absorbed by the growing medium by capillarity with an upward movement, and the drained nutrient solution was recirculated according to a closed-cycle management approach. Instead, in DI systems, the nutrient solution was delivered at the top of the growing medium and moved downward by gravity, and the drained nutrient solution was not recirculated but was managed according to an open-cycle management scheme.

### Crop and growing system management

Bok choy was seeded in rockwool cubes (2.5 × 2.5 × 4 cm, Grodan, Roermond, The Netherlands) on 24 October 2023 and 23 January 2024, for the fall and spring cycles, respectively, and was transplanted into each modular growing system 17-18 days after seeding, on 9 November 2023 and 9 February 2024, respectively.

As described by Poudel and Di Gioia (4), bok choy plants were nourished with a standard modified Sonneveld nutrient solution. During the seedling production phase, a half-strength nutrient solution was applied, while starting at planting, the same nutrient solution was applied to all the soilless growing systems. The nutrient solution was prepared using reverse-osmosis water and greenhouse-grade water-soluble fertilizers such as calcium nitrate, ammonium nitrate, potassium nitrate, potassium sulfate, magnesium sulfate, monopotassium phosphate, Fe-EDTA, Mn-EDTA, boric acid, copper sulfate, and sodium molybdate. The pH and electrical conductivity (EC) of the nutrient solution were checked upon mixing the nutrient solution and were monitored weekly using a portable pH-EC meter (HI991300, Hanna Instruments Inc., Woonsocket, RI, USA) to keep pH (5.8-6.2) and EC (1.8-2.2 dS/m) within the optimal range in both experiments. The same pH-EC meter was also used to measure the nutrient solution temperature. The nutrient solution of each modular soilless setup was recirculated in a closed system, except for the DI modules, which were managed as open-cycle systems. Adjustments of pH and EC were made by topping up the recirculating nutrient solution to replenish the water consumed using fresh nutrient solution, and when needed, pH adjustments were made using small quantities of pH-Up (General Hydroponics, USA).

Bok choy plants were harvested on 15 December 2023 and 15 March 2024, at 36 and 35 days after planting in the fall-winter and winter-spring cycles, respectively. During the two crop cycles, the average air temperature in the greenhouse was maintained around 22°C-23°C. Relative humidity ranged between 30% and 85% between the two growing cycles.

### Nutrient solution and bok choy sampling

After the bok choy was transplanted, one nutrient solution sample was collected from each of the 15 modular soilless systems weekly. The nutrient solution from the DWC and KR systems was collected directly from the reservoir. The NFT nutrient solution was sampled from the drain, which flowed back into the reservoir. The nutrient solution from the DI and EF systems was collected from the drain immediately after the system had been fertigated. Sterile bottles, autoclaved and wrapped in aluminum foil, were used to collect nutrient solution samples from each system. At each sampling point, the foil was removed, and the bottle was dipped into the nutrient solution without allowing contact between the solution and the operator’s gloves. Gloves were disinfected with ethanol between samples to minimize the chances of cross-contamination. After all samples were collected, they were transported from the greenhouse to the lab for analysis within 1 h of collection. At the end of the two growing cycles, one bok choy plant was randomly selected from each system and harvested using disinfected shears. Twenty-five grams of outer leaves were removed and placed into a sterile Whirl-Pak bag (Whirl-Pak, Pleasant Prairie, WI).

### Enumeration of aerobic bacteria

The bok choy leaves were placed in a sterile Whirl-Pak bag (Whirl-Pak) with 225 mL of sterile 1× phosphate-buffered saline and homogenized in a stomacher for 1 min at 230 RPM. The nutrient solution and homogenate from the leaves were vortexed, serially diluted, and plated in duplicate onto APC Petrifilms (3M, Hutchinson, MN) in the fall growing cycle. In the spring, SPCA was used for aerobic bacteria due to a shortage of APC Petrifilms. APC Petrifilms and SPCA plates were incubated at 35°C for 48 ± 2 h and enumerated to determine the concentration of aerobic mesophilic bacteria per milliliter of nutrient solution or gram of bok choy.

### DNA extraction

For each soilless system, 100 mL of nutrient solution from each of the biological replicates was combined to create a composite sample representative of a given system at a given sampling time point. The composite samples were filtered through 0.45 µm Nalgene Rapid-Flow Sterile Disposable Filters (Thermo Fisher Scientific, Waltham, MA, USA). DNA was extracted directly from the filters using the DNeasy PowerWater Kit (Qiagen, Hilden, Germany) according to the manufacturer’s instructions.

To assess potential contamination introduced during DNA extraction, negative extraction controls consisting of nuclease-free water processed through the extraction kit were included. In addition, the ZymoBIOMICS Microbial Community Standard (Zymo Research, Irvine, CA, USA) was processed in parallel as a positive control to verify extraction efficiency.

### 16S rRNA V4 amplicon sequencing

Extracted DNA was used to prepare libraries in the One Health Microbiome Center Research Collaboratory using their standard protocols (62). Briefly, the 16S rRNA V4 region was amplified using the KAPA HiFi HotStart mastermix and 515F (GTGYCAGCMGCCGCGGTAA) and 806R (GGACTACNVGGGTWTCTAAT) primers with partial overhangs for the i7 and i5 adapters (63). Primary qPCRs were conducted in 10 µL reactions with KAPA HiFi hot start enzyme and SYBR Green to monitor amplification in real-time, ensure successful amplification of samples, and monitor for formation of primer dimers and/or amplification of contaminant DNA in controls. Samples were subsequently diluted 100× before indexing with unique dual 10 nt indexes based on the Illumina Tagmentation Sets A-D. Resulting indexed libraries were quantified with PicoGreen and pooled in equimolar concentrations. The library was size-selected by gel purification followed by Ampure bead cleanup using a 0.6× ratio. The DNA was sequenced on an Illumina NextSeq 2000 with XLEAP P1 600-cycle reagents, generating paired-end reads of approximately 270 bp after demultiplexing. Primer sequences were removed, allowing for an error rate of 0.15 using Cutadapt/QIIME2 version 2023.5, discarding any read without a valid primer sequence on both ends.

### Sequence data analyses

Demultiplexed Illumina paired-end reads were processed using the DADA2 pipeline (v.1.26.0) in R. Reads were filtered and trimmed using the filterAndTrim() function with truncation lengths of 240 bp for both forward and reverse reads and a maximum expected error threshold of 2. All other parameters were set to default values.

Error rates were learned from the filtered reads using learnErrors function with default settings. ASVs were inferred using the dada function (pool = FALSE), and paired-end reads were merged using mergePairs function with default parameters. Following merging, only sequences between 251 and 256 bp were retained, corresponding to the expected length of the V4 region of the 16S rRNA gene. Chimeras were removed using removeBimeraDenovo and consensus method.

A sequence table was constructed using makeSequenceTable function. Taxonomy was assigned with assignTaxonomy function using the SILVA v138.2 reference database, followed by species-level refinement with addSpecies. ASVs annotated as chloroplast or mitochondria were removed before downstream analyses.

### Statistical analysis

All statistical analyses were completed in R version 4.4.1. A linear model was used to assess the effects of system, temperature, and pH on log-transformed aerobic mesophilic bacteria concentrations. Separate models were run for each growing cycle, and interaction terms were included to evaluate whether the relationships between environmental variables and APC differed by growing cycle. Temperature and pH were treated as continuous covariates, and system was treated as a categorical fixed effect. When system effects were significant, post hoc pairwise comparisons were conducted using Tukey’s Honestly Significant Difference to identify differences between individual systems. Aitchison distances were computed from CLR-transformed ASV data. Because CLR transformation cannot be applied to zeros, raw ASV counts were first processed with the count zero multiplicative (CZM) method using cmultRepl using CZM method, and the resulting values were converted to relative abundances. ASVs with extremely low abundance were removed by retaining only those taxa with a minimum relative abundance > 1 × 10⁻⁶ across all samples prior to CLR transformation. Pairwise community differences based on Aitchison distance were then evaluated using the pairwiseAdonis2 function (pairwiseAdonis package v.0.4.1). Differentially abundant ASVs between system groups were identified using the aldex.clr and aldex.ttest functions from the ALDEx2 v.1.30.0 package. ASV reads were rarified to a depth of 1,500 reads and Shannon diversity was computed and used to assess diversity over time, between systems, and within systems.

Core taxa analysis code was adapted from Rolon et al. (64). Core taxa were defined as ASVs detected in all tested samples within a system with no required minimum abundance. A phyloseq object was constructed from the ASV table, taxonomy table, and sample metadata, and then subset by system (DI, DWC, EF, KR, NFT) and growing cycle (spring, fall). Within each system-growing cycle subset, ASVs with a total count of zero across all samples were removed. Mean relative abundance for each ASV in each system-growing cycle combination was calculated as the average relative abundance across all samples in that subset.

## Data Availability

All code used for sequence processing, statistical analyses, and figure generation is publicly available in a dedicated GitHub repository (https://github.com/abywats717/Bok_Choy_Soilless_Farming). Raw demultiplexed sequence data have been deposited in the NCBI Sequence Read Archive under BioProject accession number PRJNA1366713.

## SUPPLEMENTAL MATERIAL

Supplemental material aem.01167-26-s0001.docx Table S1 and Fig. S1 to S11.

ASM does not own the copyrights to Supplemental Material that may be linked to, or accessed through, an article. The authors have granted ASM a non-exclusive, world-wide license to publish the Supplemental Material files. Please contact the corresponding author directly for reuse.
