# Supplementary material for: Soilless farming system design impacts the diversity and composition of microbiota
Source: Appl Environ Microbiol. 2026 Aug 4;92(9):e01167-26. doi: 10.1128/aem.01167-26 (PMC13599518; doi:10.1128/aem.01167-26)
Supplement: Supplementary file 1 [file aem.01167-26-s0001.docx]

**Supplemental Material**

**TABLE S1** Nutrient solution average temperatures and pH values with standard deviations for each system.

| System^a^ | Growing Cycle | Average Temperature (°C) | Temperature standard deviation (°C) | Average pH | pH standard deviation |
| --- | --- | --- | --- | --- | --- |
| DI | Fall | 22.5 | 2.3 | 5.9 | 0.3 |
|  | Spring | 23.3 | 1.5 | 7.1 | 0.8 |
| DWC | Fall | 21.4 | 2.6 | 5.9 | 0.2 |
|  | Spring | 22.6 | 1.2 | 6.7 | 0.7 |
| EF | Fall | 21.3 | 2.4 | 6.0 | 0.3 |
|  | Spring | 21.3 | 2.4 | 6.0 | 0.3 |
| KR | Fall | 21.3 | 2.4 | 6.0 | 0.3 |
|  | Spring | 22.6 | 0.9 | 6.7 | 0.6 |
| NFT | Fall | 21.3 | 2.4 | 6.0 | 0.3 |
|  | Spring | 22.9 | 1.5 | 6.8 | 0.8 |
|  |  |  |  |  |  |

^a^DI, drip irrigation; DWC, deep water culture, EF, ebb and flow, KR, Kratky, NFT, nutrient film technique.

**FIG S1** Average aerobic plate count (APC) on the outer leaves of the harvested bok choy at the end of each growing cycle. DI, drip irrigation; DWC, deep water culture, EF, ebb and flow, KR, Kratky, NFT, nutrient film technique.

**FIG S2** Occupancy abundance relationship of bacterial genera in the drip irrigation (DI) system during fall growing cycle. Each point represents a bacterial genus, plotted by mean relative abundance (log_10_-transformed) and occupancy across samples within the system. Genera with higher occupancy and abundance indicate taxa that were more consistently detected across samples during the sampling period.

**FIG S3** Occupancy abundance relationship of bacterial genera in the drip irrigation (DI) system during spring growing cycle. Each point represents a bacterial genus, plotted by mean relative abundance (log_10_-transformed) and occupancy across samples within the system. Genera with higher occupancy and abundance indicate taxa that were more consistently detected across samples during the sampling period.

**FIG S4** Occupancy abundance relationship of bacterial genera in the deep water culture (DWC) system during fall growing cycle. Each point represents a bacterial genus, plotted by mean relative abundance (log_10_-transformed) and occupancy across samples within the system. Genera with higher occupancy and abundance indicate taxa that were more consistently detected across samples during the sampling period.

**FIG S5** Occupancy abundance relationship of bacterial genera in the deep water culture (DWC) system during spring growing cycle. Each point represents a bacterial genus, plotted by mean relative abundance (log_10_-transformed) and occupancy across samples within the system. Genera with higher occupancy and abundance indicate taxa that were more consistently detected across samples during the sampling period.

**FIG S6** Occupancy abundance relationship of bacterial genera in the ebb and flow (EF) system during fall growing cycle. Each point represents a bacterial genus, plotted by mean relative abundance (log_10_-transformed) and occupancy across samples within the system. Genera with higher occupancy and abundance indicate taxa that were more consistently detected across samples during the sampling period.

**FIG S7** Occupancy abundance relationship of bacterial genera in the ebb and flow (EF) system during spring growing cycle. Each point represents a bacterial genus, plotted by mean relative abundance (log_10_-transformed) and occupancy across samples within the system. Genera with higher occupancy and abundance indicate taxa that were more consistently detected across samples during the sampling period.

**FIG S8** Occupancy abundance relationship of bacterial genera in the Kratky (KR) system during fall growing cycle. Each point represents a bacterial genus, plotted by mean relative abundance (log_10_-transformed) and occupancy across samples within the system. Genera with higher occupancy and abundance indicate taxa that were more consistently detected across samples during the sampling period.

 

**FIG S9** Occupancy abundance relationship of bacterial genera in the Kratky (KR) system during spring growing cycle. Each point represents a bacterial genus, plotted by mean relative abundance (log_10_-transformed) and occupancy across samples within the system. Genera with higher occupancy and abundance indicate taxa that were more consistently detected across samples during the sampling period.

 

**FIG S10** Occupancy abundance relationship of bacterial genera in the nutrient film technique (NFT) system during fall growing cycle. Each point represents a bacterial genus, plotted by mean relative abundance (log_10_-transformed) and occupancy across samples within the system. Genera with higher occupancy and abundance indicate taxa that were more consistently detected across samples during the sampling period.

 

**FIG S11** Occupancy abundance relationship of bacterial genera in the nutrient film technique (NFT) system during spring growing cycle. Each point represents a bacterial genus, plotted by mean relative abundance (log_10_-transformed) and occupancy across samples within the system. Genera with higher occupancy and abundance indicate taxa that were more consistently detected across samples during the sampling period.
